# Romidepsin for the treatment of relapsed/refractory peripheral T-cell lymphoma: pivotal study update demonstrates durable responses

**DOI:** 10.1186/1756-8722-7-11

**Published:** 2014-01-23

**Authors:** Bertrand Coiffier, Barbara Pro, H Miles Prince, Francine Foss, Lubomir Sokol, Matthew Greenwood, Dolores Caballero, Franck Morschhauser, Martin Wilhelm, Lauren Pinter-Brown, Swaminathan Padmanabhan Iyer, Andrei Shustov, Tina Nielsen, Jean Nichols, Julie Wolfson, Barbara Balser, Steven Horwitz

**Affiliations:** 1 Hospices Civils de Lyon, Lyon, France; 2 Kimmel Cancer Center, Thomas Jefferson University, Philadelphia, PA, USA; 3 Peter MacCallum Cancer Centre and University of Melbourne, Melbourne, Australia; 4 Yale Cancer Center, New Haven, CT, USA; 5 Moffitt Cancer Center, Tampa, FL, USA; 6 Royal North Shore Hospital, Sydney, Australia; 7 Hospital Universitario de Salamanca, Salamanca, Spain; 8 Hôpital Claude Huriez, CHRU de Lille, Lille, France; 9 Klinikum Nürnberg Nord, Nürnberg, Germany; 10 UCLA Medical Center, Los Angeles, CA, USA; 11 The Methodist Hospital, Houston, TX, USA; 12 University of Washington, Seattle, WA, USA; 13 Celgene Corporation, Summit, NJ, USA; 14 JNichols LLC, Swampscott, MA, USA; 15 Veristat, LLC, Holliston, MA, USA; 16 Memorial Sloan-Kettering Cancer Center, New York, NY, USA

**Keywords:** Romidepsin, Relapsed/refractory peripheral T-cell lymphoma, Duration of response

## Abstract

**Background:**

Romidepsin is a structurally unique, potent, bicyclic class 1 selective histone deacetylase inhibitor approved by the US Food and Drug Administration for the treatment of patients with cutaneous T-cell lymphoma who have received ≥ 1 prior systemic therapy and patients with peripheral T-cell lymphoma (PTCL) who have received ≥ 1 prior therapy. Approval for PTCL was based on results (n = 130; median follow-up, 13.4 months) from the pivotal study of romidepsin for the treatment of relapsed/refractory PTCL. The objective is to present updated data (median follow-up, 22.3 months) and to characterize patients who achieved long-term responses (≥ 12 months) to romidepsin.

**Methods:**

Patients with PTCL who relapsed from or were refractory to ≥ 1 prior systemic therapy received romidepsin 14 mg/m^2^ as a 4-hour intravenous infusion on days 1, 8, and 15 every 28 days for up to 6 cycles; patients with response or stable disease could continue romidepsin beyond 6 cycles. The primary endpoint was rate of confirmed/unconfirmed complete response (CR/CRu) determined by an Independent Review Committee. Secondary endpoints included objective response rate (ORR) and duration of response (DOR). For patients who achieved CR/CRu, baseline characteristics by DOR (≥ 12 vs < 12 months) were examined.

**Results:**

The ORR to romidepsin was 25%, including 15% with CR/CRu. The median DOR for all responders was 28 months (range, < 1-48+) and was not reached for those who achieved CR/CRu. Patients with lack of response or transient response to prior therapy achieved durable responses with romidepsin. Of the 19 patients who achieved CR/CRu, 10 had long-term (≥ 12 months) responses; none of the baseline characteristics examined—including heavy pretreatment, response to prior therapy, or advanced disease—precluded long-term responses to romidepsin. With a median progression-free survival of 29 months, patients who achieved CR/CRu for ≥ 12 months had significantly longer survival vs those with CR/CRu for < 12 months or < CR/CRu. Extended treatment and longer follow-up did not affect the reported safety profile of romidepsin.

**Conclusions:**

Treatment with romidepsin leads to highly durable responses in a subset of patients with relapsed/refractory PTCL, with responses ongoing as long as 48 months.

**Trial registration:**

NCT00426764

## Background

Peripheral T-cell lymphoma (PTCL) is an aggressive, uncommon form of non-Hodgkin lymphoma (NHL) that is typically associated with a poor prognosis [1-3]. PTCL comprises many subtypes that vary in morphology, biology, and prognosis [1,2]. The most common subtypes globally are PTCL–not otherwise specified (NOS), angioimmunoblastic T-cell lymphoma (AITL), and anaplastic large cell lymphoma (ALCL) [2]. In western countries, PTCL accounts for 15% to 20% of aggressive lymphomas and 5% to 10% of NHL diagnoses. The prevalence is higher in Asia, with approximately 15% to 20% of all lymphomas classified as PTCL or natural killer/T-cell lymphoma [2,4-6]. Some of this variation may be a result of exposure or genetic susceptibility to pathogenic agents such as human T-lymphotropic virus-1 and Epstein-Barr virus in Asia [2,5,7].

There is no current standard of care for patients with most subtypes of PTCL, and no agents have been approved specifically for use as first-line treatment of PTCL [3,8]. In the first line, most patients receive induction chemotherapy regimens derived from studies of B-cell lymphomas, most commonly anthracycline-containing regimens such as CHOP (cyclophosphamide, doxorubicin, vincristine, prednisone) [1-3,8-11]. Although most patients achieve a response with induction chemotherapy, responses are typically brief and many patients experience relapse or become refractory to treatment [1-3,9,10]. The role of stem cell transplantation (SCT) for patients with PTCL is yet to be clearly determined and, currently, only a minority (< 20%) of patients undergo SCT [9-11]. Many patients with PTCL who receive SCT experience disease relapse after transplantation [12].

Romidepsin—a structurally unique, potent, bicyclic class 1 selective histone deacetylase inhibitor [13-15]—is approved for the treatment of both patients with cutaneous T-cell lymphoma who have received ≥ 1 prior systemic therapy and patients with PTCL who have received ≥ 1 prior therapy [16]. Approval in PTCL was primarily based on results from a phase 2, single-arm, open-label study in relapsed/refractory PTCL (GPI-06-0002) [17].

For romidepsin, data from GPI-06-0002 (n = 130) based on an October 2010 cutoff (median follow-up, 13.4 months) were presented in the package insert [16] and published manuscript [17] and include a 25% objective response rate (ORR), 15% confirmed/unconfirmed complete response (CR/CRu) rate, and median duration of response (DOR) of 17 months [17]. As of the October 2010 data cutoff, 17 of 19 patients who had experienced CR/CRu had not progressed [17]. The objective of this manuscript is to present updated GPI-06-0002 efficacy data and characteristics of patients who achieved long-term responses (≥ 12 months) based on a more recent Independent Review Committee (IRC) assessment (data cutoff, December 31, 2011; median follow-up, 22.3 months).

## Results

### Patient characteristics

A total of 131 patients were enrolled; 130 had histologically confirmed PTCL by central review, and 1 had a diagnosis of diffuse large B-cell lymphoma by central review and was excluded from the baseline measurements and efficacy assessments. Patient demographics and baseline characteristics were previously described in detail (n = 130) [17]. Briefly, most patients had an Eastern Cooperative Oncology Group (ECOG) performance status of 1 to 2 (64%), International Prognostic Index ≥ 2 (76%), and stage III or IV disease (70%). Thirty-six patients (28%) had bone marrow involvement. Patients had received a median of 2 (range, 1–8) prior systemic therapies; 37% of patients had received ≥ 3 therapies, and 16% had received prior autologous SCT. The most common PTCL subtypes were PTCL–NOS, AITL, and anaplastic lymphoma kinase (ALK)-1–negative ALCL; baseline characteristics were similar across these common PTCL subtypes.

### Efficacy

#### Response rates

Response rates as assessed by the IRC were unchanged compared with the primary publication [17]: ORR of 25% (33 of 130), including CR/CRu in 15% of patients (19 of 130). At a median follow-up of 22.3 months, all patients received a median of 2 treatment cycles (range, < 1–54), whereas patients who achieved a response (CR/CRu or partial response [PR]; n = 33) received a median of 8 treatment cycles (range, < 1–54) and patients who achieved a CR/CRu (n = 19) received a median of 19 treatment cycles (range, 2–54). Response rates were similar across the 3 most common PTCL subtypes (Table 1), and no significant differences in ORR or rates of CR/CRu were observed.

**Table 1:** Overall Response Rates (IRC)

**Best response, n (%)**	**PTCL-NOS**	**AITL**	**ALK-1–Negative ALCL**
	**(n = 69)**	**(n = 27)**	**(n = 21)**
ORR	20 (29)	8 (30)	5 (24)
CR/CRu	10 (14)	5 (19)	4 (19)
PR	10 (14)	3 (11)	1 (5)
SD	16 (23)	9 (33)	5 (24)
Disease control (ORR + SD90)	34 (49)	12 (44)	8 (38)
PD/NE^a^	33 (48)	10 (37)	11 (52)

*Abbreviations*: *AITL* angioimmunoblastic T-cell lymphoma, *ALCL* anaplastic large cell lymphoma, *ALK* anaplastic lymphoma kinase, *CR*/*CRu* confirmed/unconfirmed complete response, *IRC* Independent Review Committee, *NE* not evaluable, *ORR* objective response rate, *PD* progressive disease, *PR* partial response, *PTCL*-*NOS* peripheral T-cell lymphoma–not otherwise specified, *SD* stable disease, *SD90* SD for ≥ 90 days.

^a^Insufficient efficacy data to determine response because of early termination.

#### Durability of responses

**Figure 1:**
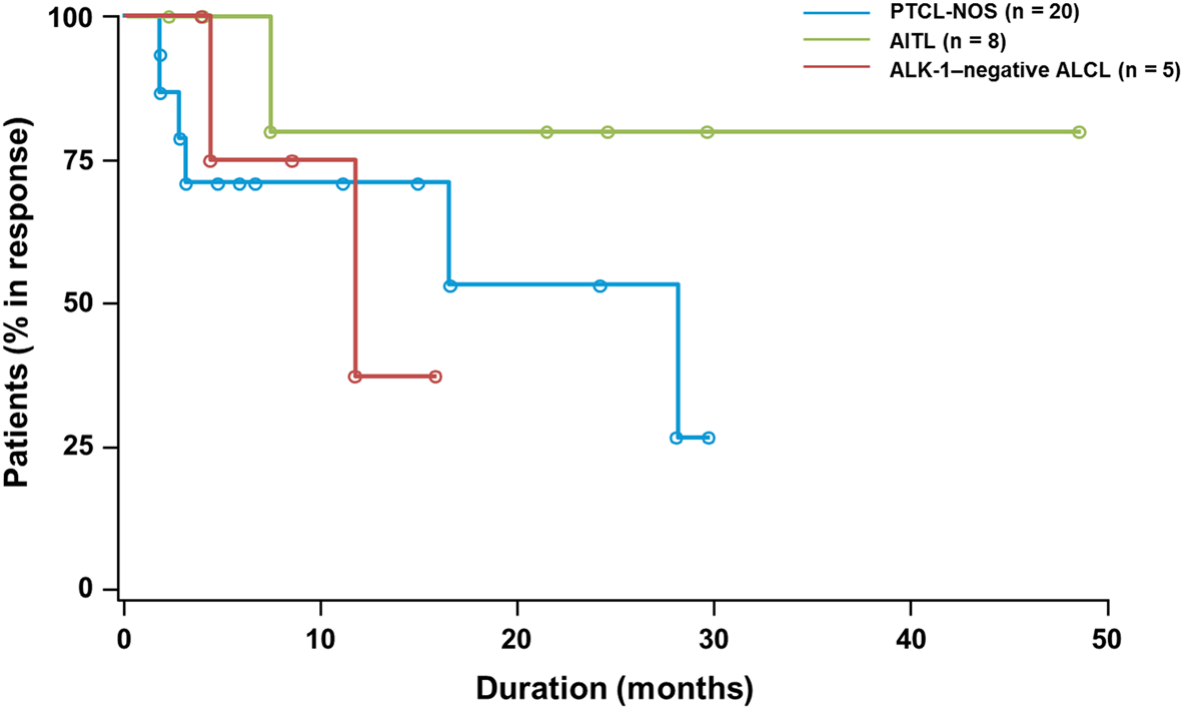
**Durations of response for the 3 most common subtypes of PTCL in patients who achieved a response (CR or PR).** ◦ Indicates a censored patient.

Most responses were noted at the first response assessment (2 months), and the median time to response as assessed by the IRC was 1.8 months (range, 1.4-5.3 months). The median DOR for all patients who achieved a response by IRC (n = 33) was 28 months (range, < 1-48+) and had not been reached (range, < 1-48+) for those who achieved CR/CRu (n = 19). One patient with a reported DOR < 1 month discontinued treatment to receive SCT after the first response assessment of CR. Of the 19 patients who achieved CR/CRu, 53% had a DOR ≥ 12 months and 32% had a DOR ≥ 24 months. Responses were durable across the 3 most common PTCL subtypes (Figure 1), and no statistically significant differences in DOR were observed. For patients with progressive disease (PD) to their last prior therapy (n = 49), the median DOR on romidepsin had not yet been reached for all patients who achieved a response (n = 14) or for patients wxho achieved CR/CRu (n = 9).

**Figure 2:**
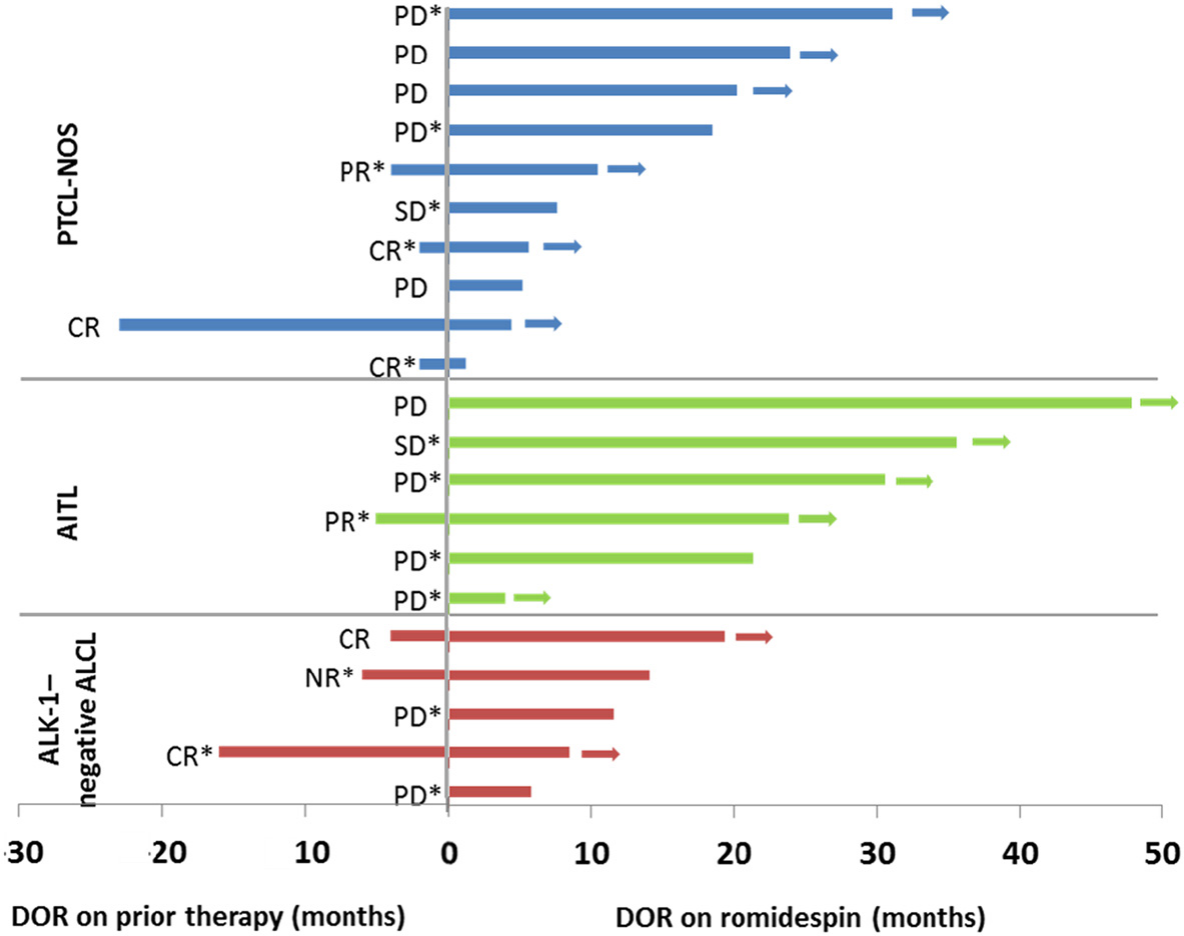
**Durations of response for patients who achieved CR/CRu on romidepsin (investigator assessed; n = 21).** Only investigators assessed DOR for prior therapy (for patients with PR or CR on prior therapy). Arrows indicate that the patient was censored while in response. * Indicates that the patient received combination chemotherapy as the last prior therapy. NR indicates that the response to prior therapy was not reported.

Patients with a lack of response (n = 13) or transient response (n = 8; median DOR, 4.5 months [range, 2–23 months]) to prior therapy (most commonly CHOP [n = 7], GVD [gemcitabine, vinorelbine, doxorubicin; n = 2], ICE [ifosfamide, carboplatin, etoposide; n = 2], ESHAP [etoposide, methylprednisolone, high-dose cytarabine, cisplatin; n = 2], or pralatrexate [n = 2]), by investigator assessment, were able to achieve durable CR/CRu to romidepsin (median DOR, 14 months [range 1-48+ months]) across the 3 most common PTCL subtypes (Figure 2). Patients who achieved CR/CRu with a reported DOR < 12 months (investigator assessment) discontinued treatment for the following reasons: adverse event (AE; n = 2), patient or investigator decision to stop therapy while in CR, physician decision, and SCT (each n = 1).

#### Analysis of long-term responders

**Figure 3:**
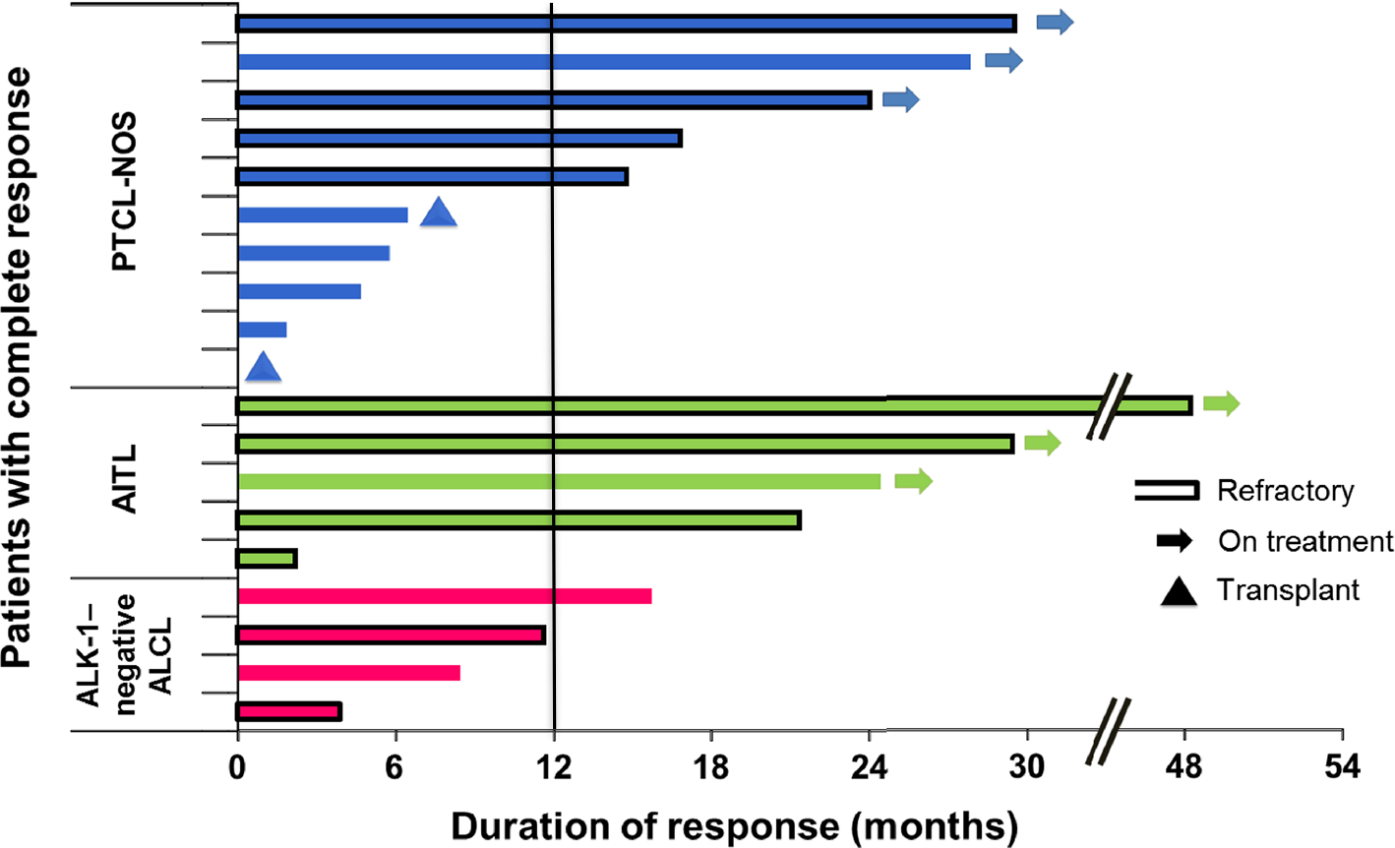
**Durations of response for patients who achieved CR/CRu on romidepsin (IRC).** The vertical line separates the 10 patients with long-term responses (duration ≥ 12 months). Of the 2 patients that received stem cell transplant after romidepsin, type of transplant was autologous for 1 patient, and not reported for 1 patient.

Of the 19 patients who achieved CR/CRu (Figure 3), 16 (84%) had not experienced PD per the IRC at a median follow-up of 25.8 months. For those 16 patients, the DOR at time of censoring ranged from < 1 month to 48+ months. Ten of 19 patients who achieved CR/CRu were considered long-term responders (responses ≥ 12 months). Of these 10 patients, 1 had IRC-confirmed PD; 3 were censored as a result of investigator-assessed PD (n = 1), physician decision (n = 1), or AE (n = 1); and 6 continued romidepsin treatment for ≥ 2 years (received 25, 27, 29, 36, 36, and 53 cycles as of the data cutoff). Nine of the 19 patients who achieved CR/CRu had a reported DOR < 12 months. Two had IRC-confirmed PD, and 7 were censored because investigator-assessed PD (n = 1), SCT (n = 2), physician decision (n = 2), or patient decision (n = 2). None of the baseline characteristics examined—including heavy pretreatment (≥ 3 prior therapies), response to prior therapy, or advanced disease—precluded long-term responses to romidepsin (Table 2).

**Table 2:** Key Baseline Characteristics by Duration of CR/CRu

	**CR/CRu ≥ 12 months (n = 10)**	**CR/CRu < 12 months (n = 9)**	***P *** **value**
Male sex, n (%)	7 (70)	5 (56)	.65
Median age, years (range)	61.5 (47–78)	57.0 (37–74)	.43
ECOG performance status, n (%)			
0	3 (30)	4 (44)	.57
1	6 (60)	3 (33)	
2	1 (10)	2 (22)	
IPI score, n (%)			
< 2	2 (20)	1 (11)	1.00
≥ 2	8 (80)	8 (89)	
Prior systemic therapies, n (%)			
1	2 (20)	3 (33)	.38
2	3 (30)	3 (33)	
3	0	2 (22)	
4	3 (30)	1 (11)	
> 4	2 (20)	0	
Best response to most recent prior therapy, n (%)			
CR/CRu/PR	2 (20)	6 (67)	.07
< PR	8 (80)	3 (33)	
Disease stage at diagnosis, n (%)			
I/II	0 (0)	4 (44)	.03
III/IV	10 (100)	5 (56)	
PTCL subtype, n (%)			
PTCL-NOS	5 (50)	5 (56)	.34
AITL	4 (40)	1 (11)	
ALK-1–negative ALCL	1 (10)	3 (33)	
Bone marrow involvement, n (%)	5 (50)	2 (22)	.35
Elevated LDH, n (%)	5 (50)	8 (89)	.14
Platelet count x 10^9^/L, median (range)	199 (101–307)	172 (99–649)	.42

*Abbreviations*: *AITL* angioimmunoblastic T-cell lymphoma, *ALCL* anaplastic large cell lymphoma, *ALK* anaplastic lymphoma kinase, *CR* complete response, *CR*/*CRu* confirmed/unconfirmed complete response, *ECOG* Eastern Cooperative Oncology Group, *IPI* International Prognostic Index, *LDH* lactate dehydrogenase, *NOS* not otherwise specified, *PR* partial response, *PTCL* peripheral T-cell lymphoma.

**Figure 4:**
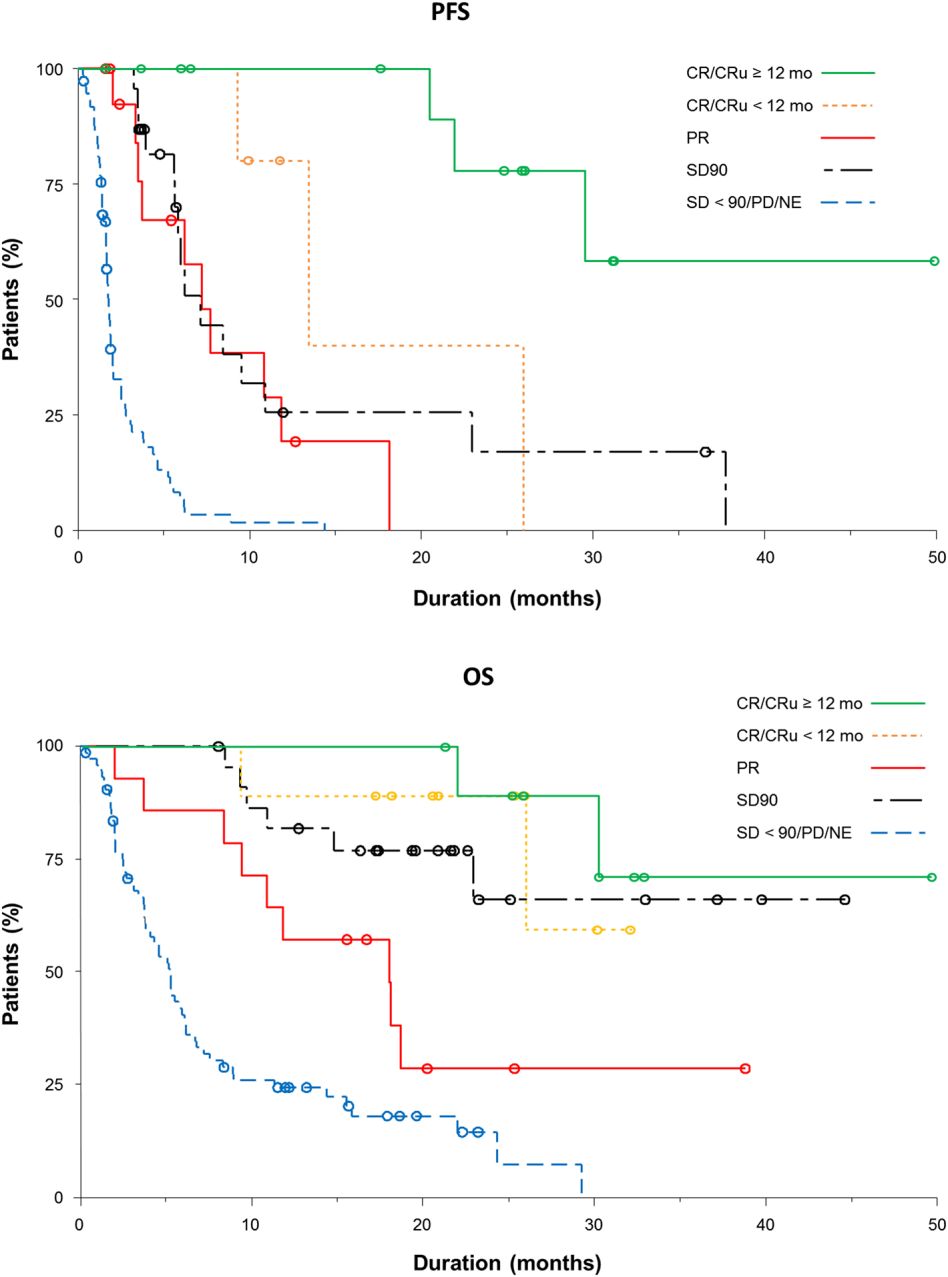
Survival (PFS, OS) by quality of response to romidepsin.

Achievement of CR/CRu was associated with prolonged progression-free survival (PFS; median 29 months) and overall survival (OS; median not reached) compared with patients with a response < CR/CRu. Furthermore, achievement of CR/CRu at ≥ 12 months was associated with prolonged PFS and OS compared with achievement of CR/CRu at < 12 months (Figure 4, Table 3; P < .05). Patients who achieved PR or stable disease (SD) for ≥ 90 days (SD90) had similar long-term outcomes. For all patients, median PFS and OS were 4 months and 11.3 months, respectively.

**Table 3:** Survival (PFS, OS) by Quality of Response to Romidepsin

	**Objective responders**	**CR/CRu**	**CR/CRu**	**PR**	**SD90**	**SD < 90/ PD/NE**
		**≥ 12 months**	**< 12 months**			
	**(CR/CRu + PR; n = 33)**	**(n = 10)**	**(n = 9)**	**(n = 14)**	**(n = 23)**	**(n = 74)**
Median OS, months (range)	30	NR	NR	18	NR	5
	(2.0-49.5)	(21.2-49.5)	(9.2-32.0)	(2.0-38.8)	(8.1-44.6)	(0.3-29.3)
Median PFS, months, (range)	20	29	13	7	7	2
	(1.6-49.8)	(17.6-49.8)	(1.6-26.0)	(1.9-18.1)	(3.3-37.7)	(0.3-14.4)

*Abbreviations*: *CR*/*CRu* confirmed/unconfirmed complete response, *NR* not reached, *OS* overall survival, *PD* progressive disease, *PFS* progression-free survival, *PR* partial response, *SD* stable disease, *SD90* SD ≥ 90 days.

### Safety

**Figure 5:**
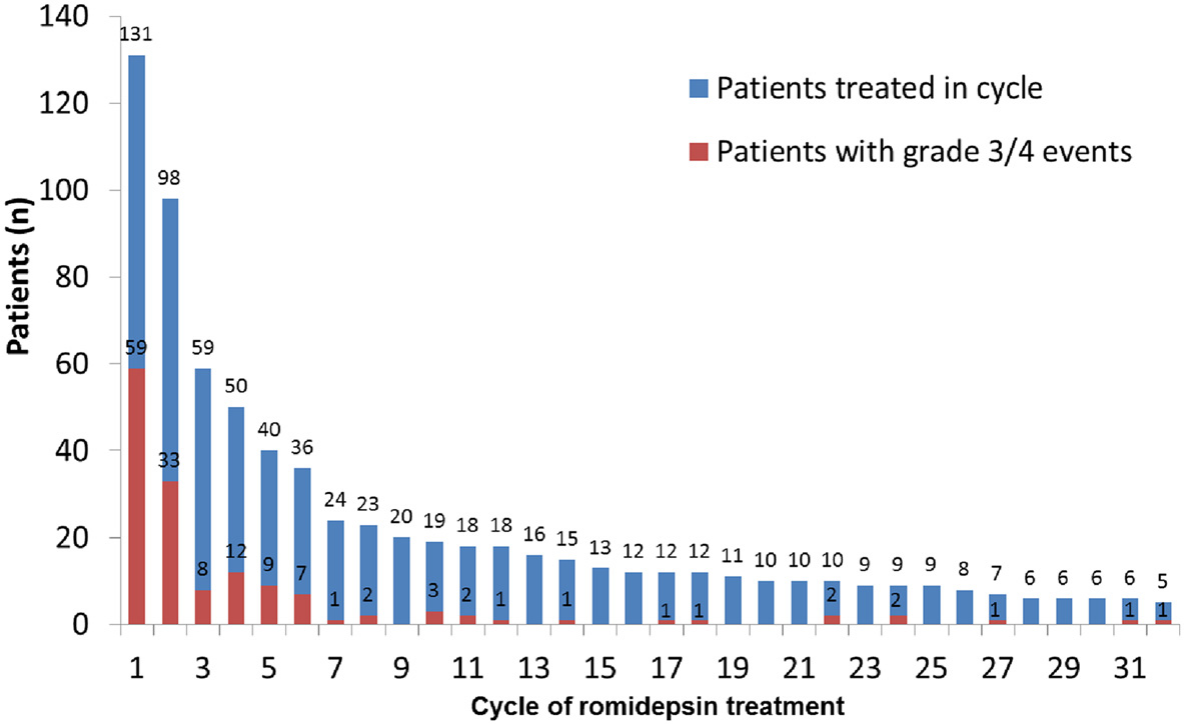
**Incidence of grade ≥ 3 AEs by treatment cycle.** Cycles in which no grade ≥ 3 AEs were reported are not included.

As previously reported, the most common AEs included gastrointestinal disturbances, hematologic abnormalities, asthenic conditions, and infections (all types pooled) [17]. Reported electrocardiogram (ECG) abnormalities were uncommon, with no concurrent symptoms of syncope or other cardiac AEs at the time of reported ECG abnormality and no clinically significant changes in QT intervals across treatment cycles were found [17]. The AE profile was similar across the 3 most common PTCL subtypes. Longer treatment duration did not affect the safety profile of romidepsin. Grade ≥ 3 AEs occurred with the highest incidence during cycles 1 to 2 (Figure 5). After cycle 18, ≤ 10 patients remained on treatment, and most grade ≥ 3 AEs reported were from 1 patient with grade ≥ 3 vomiting, cellulitis, deep vein thrombosis, and/or constipation in cycles 22, 24, 27, 31, and 32. In addition, one patient had grade ≥ 3 pyrexia in cycle 22 and one patient had grade ≥ 3 pneumonia in cycle 24.

## Discussion

Since 2009, 3 agents have been approved in the United States for patients with relapsed/refractory PTCL [16,18,19]. Pralatrexate, a folate analogue, is approved for the treatment of patients with relapsed or refractory PTCL [18] based on a single-arm phase 2 study showing 29% ORR, including 11% CR (n = 109) and a median DOR of 10.1 months [20]. Brentuximab vedotin, a CD30-directed antibody-drug conjugate, is approved for the treatment of patients with systemic ALCL after failure of ≥ 1 prior multiagent chemotherapy regimen [19]. ALCL is a common subtype of PTCL (approximately 25% of cases in North America) that is uniformly CD30+ [2,21]. Approval was based on an 86% ORR, including a 57% CR (n = 58) [21] and a median DOR of 13.2 months [22]. In patients with relapsed/refractory PTCL, romidepsin demonstrated a 25% ORR, including 15% CR/CRu and a median DOR of 28 months for all responders. Responses were rapid with a median time to objective response of 1.8 months. Long-lasting responses occurred across all major PTCL subtypes and in patients refractory to their last prior therapy.

Romidepsin has demonstrated comparable efficacy across the 3 most common PTCL subtypes. Although pralatrexate has general approval for the treatment of PTCL, based on results from the pivotal study in relapsed/refractory PTCL [20], National Comprehensive Cancer Network guidelines report that pralatrexate has demonstrated limited activity in AITL [8]. Brentuximab vedotin has demonstrated considerable activity in ALCL [21], and responses have also been seen in CD30+ cases of other NHL subtypes (including AITL) [23]; however, its utility in non-CD30+ PTCLs is unknown.

More than one-half of the patients with relapsed/refractory PTCL who achieved CR/CRu on romidepsin experienced long-term responses (≥ 12 months). Complete responses were achieved in patients with typically poor prognostic factors, and none of the examined patient or disease characteristics predicted failure to achieve long-term remission. Achievement of CR/CRu was associated with prolonged survival, and achievement of SD90 led to survival rates similar to the achievement of PR.

Amendments to the study protocol allowed for maintenance with 2 romidepsin doses per cycle for patients who received ≥ 12 treatment cycles and with 1 dose per cycle for patients who received ≥ 24 treatment cycles and had received 2 doses for ≥ 6 treatment cycles.

Extended dosing of romidepsin was tolerated; the most grade ≥ 3 AEs were observed in the first 2 cycles of treatment. ECG abnormalities were uncommon, and no clinically significant changes were observed across treatment cycles [17]. An early analysis of a post-marketing QT study demonstrated that romidepsin does not have a concentration-dependent effect on the QTc interval (including at exposures more than 2-fold the approved dosing), and while clinically insignificant changes in QTc were reported, these changes were attributable to antiemetic premedication [24]. Romidepsin was associated with a delayed concentration-dependent increase in heart rate with a maximum mean increase of 20 beats per minute 6 hours after the start of a 4-hour romidepsin infusion [16]. Publication of the final analysis of the QT study is ongoing.

A SEER (Surveillance and Epidemiology End Results) database analysis from 1992 to 2009 showed that, with the exception of ALCL, the incidence of common PTCL subtypes has greatly increased over the past few decades (the combined rate more than tripled from 1992 to 2009) [25]. However, over this same time period, survival times did not increase [25] as they have for patients with B-cell lymphoma [26]. Despite poor outcomes, anthracycline-based regimens continue to be commonly used for the treatment of PTCL [1-3,8-11]. With the approval of 3 novel agents since 2009 [16,18,19], it is hoped that a trend toward improved survival will begin to emerge. Current studies are evaluating the combination of these newly approved agents with chemotherapeutic regimens. Preliminary results from a phase 1B study of romidepsin in combination with CHOP for the first-line treatment of patients with PTCL (14 evaluable patients) demonstrated an ORR of 78%, including 57% CR [27], and a phase 2 extension study is ongoing. A separate phase 3 study of romidepsin + CHOP vs CHOP alone in frontline PTCL is also ongoing [28]. Preliminary results from a phase 1 study of brentuximab vedotin with CH-P (CHOP minus vincristine) for the first-line treatment of patients with higher-risk systemic ALCL and other CD30+ mature natural killer/T-cell lymphomas (n = 26) demonstrated an ORR of 100%, including 88% CR [29]. A phase 2 study of CEOP (cyclophosphamide, etoposide, vincristine, prednisone) alternating with pralatrexate for the first-line treatment of PTCL is underway [30], and a separate phase 3 study will investigate pralatrexate vs observation maintenance therapy following CHOP-based induction in patients with PTCL [31]. Long-term follow-up of these combination trials is essential to determine whether any of these combinations leads to durable responses.

## Conclusions

The results presented herein demonstrate that treatment with single-agent romidepsin leads to highly durable responses in patients with relapsed/refractory PTCL, including patients with the 3 major PTCL subtypes, patients who received several prior systemic therapies, patients with advanced disease, and, importantly, patients refractory to their last prior therapy. Patients with long-term responses to romidepsin can successfully continue on romidepsin, with or without reducing dose frequency, to maintain response at the discretion of the investigator. Whether combining romidepsin with regimens that induce higher initial response rates (eg, CHOP) will enhance the durability of these responses and lead to prolonged survival, both in relapsed/refractory patients and in those with newly diagnosed disease, warrants further investigation. Additionally, the potential for use of romidepsin as maintenance therapy after chemotherapy induction or after consolidation with high-dose chemotherapy followed by SCT should be examined, because long-term tolerability has been demonstrated.

## Methods

### Study design and eligibility criteria

GPI-06-0002 is a prospective, single-arm, open-label, international phase 2 study that was previously described in detail [17]. Briefly, eligibility criteria included adult patients with PTCL relapsed or refractory to ≥ 1 systemic therapy, adequate bone marrow and organ function, and measurable disease according to International Working Group (IWG) criteria [32] and/or measurable cutaneous disease and an ECOG performance status of 0 to 2 at enrollment. Concomitant use of drugs that could significantly prolong the QTc interval was not allowed, and patients with known significant cardiac abnormalities were excluded. Hypokalemia and hypomagnesemia can be associated with ECG abnormalities [33]; therefore, patients must have had serum potassium concentrations ≥ 3.8 mmol/L and serum magnesium concentrations ≥ 0.85 mmol/L; low levels could be corrected by supplementation to meet inclusion criteria.

Patients received romidepsin 14 mg/m^2^ (4-hour intravenous infusion) on days 1, 8, and 15 of 28-day cycles (same dose and schedule as those approved for patients with relapsed/refractory cutaneous T-cell lymphoma or PTCL [16]) for up to 6 cycles. Patients with SD or response could continue to receive treatment beyond 6 cycles at the discretion of the patient and investigator. The protocol was amended to allow for (but not mandate) maintenance dosing of romidepsin (2 doses per cycle) for patients treated for ≥ 12 cycles. Patients who initiated maintenance dosing must continue to receive ≥ 2 doses per cycle through at least cycle 24 and must have received 2 doses per cycle for ≥ 6 months prior to a reduction to 1 dose per cycle.

### Response assessments and study endpoints

The efficacy and safety assessments conducted and the response criteria used were previously described in detail [17,32]. Briefly, response assessments were performed every 2 cycles by both investigators and an IRC (composed of both radiologists and hematologic oncologists) according to the 1999 IWG criteria guidelines for response assessments for NHL (IWG-NHL) [32]. The primary endpoint was rate of CR/CRu as determined by IRC. Secondary endpoints included ORR, DOR (time from the first date of response to the date of PD or date of last study assessment), and time to disease progression by IRC and investigators’ assessments; change in ECOG performance status; tolerability; and safety. Time to response, PFS, and OS were also assessed.

### Statistical methods

This study is ongoing, but December 31, 2011, was the cutoff date for this analysis. Patients who withdrew without PD were to be followed every 2 months until PD, withdrawal from study, or start of alternate therapy. All descriptive statistical analyses were performed by using SAS statistical software version 9.2 (SAS Institute, Cary, NC). Time-to-event data were summarized by Kaplan-Meier methods.

## Competing interests

BC: Celgene Corporation. BP; honoraria: Celgene Corporation. HMP; research grant funding. FF; advisory board: Celgene Corporation. LS; consultancy, speakers bureau: Celgene Corporation; research funding: Gloucester. FM; honoraria: Celgene Corporation. LPB; consultancy: Celgene Corporation. SPI; consultancy: Celgene Corporation. AS; research funding, consultancy, honoraria: Celgene Corporation. TN; employee: Celgene Corporation. JN; employee Jan 2010 – Jul 2012: Celgene Corporation. BB; consultancy: Celgene Corporation. SH; consultancy and grant support: Celgene Corporation. CC, MG, MW, JW; The authors declare that they have no competing interests.

## Authors’ contributions

BC interpreted the data, drafted the paper, and approved all versions including the final version. BB and JW acquired and analyzed the data, critically revised the paper, and approval all versions including the final version. BP, HMP, FF, LS, FM, LPB, SPI, AS, TN, JN, SH, CC, MG, MW interpreted the data, critically revised the paper and approved all versions including the final version. All authors are responsible for the accuracy and integrity of all aspects of the manuscript.
